# Emerging systemic treatment options in meningioma

**DOI:** 10.1007/s11060-022-04148-8

**Published:** 2022-10-01

**Authors:** Maximilian J. Mair, Anna S. Berghoff, Priscilla K. Brastianos, Matthias Preusser

**Affiliations:** 1 Division of Oncology, Department of Medicine I, Medical University of Vienna, Waehringer Guertel 18-20, 1090 Vienna, Austria; 2 Christian Doppler Laboratory for Personalized Immunotherapy, Medical University of Vienna, Vienna, Austria; 3 Division of Hematology/Oncology, Department of Medicine, Massachusetts General Hospital, Harvard Medical School, Boston, MA USA; 4 Division of Neuro-Oncology, Department of Neurology, Massachusetts General Hospital, Harvard Medical School, Boston, MA USA

**Keywords:** Meningioma, Systemic treatment, Chemotherapy, Targeted therapy, Immunotherapy

## Abstract

**Purpose:**

Meningiomas are the most frequently diagnosed intracranial neoplasms. Usually, they are treated by surgical resection in curative intent. Radiotherapy and stereotactic radiosurgery are commonly applied in the adjuvant setting in newly diagnosed atypical (CNS WHO grade 2), and anaplastic (CNS WHO grade 3) meningioma, especially if gross total resection is not feasible, and in recurrent cases. Conversely, the evidence for pharmacotherapy in meningioma is scarce.

**Methods:**

The available literature of systemic treatment in meningioma was screened using PubMed, and ongoing clinical trials were explored using ClinicalTrials.gov.

**Results:**

Classical cytotoxic agents, somatostatin analogs, and antihormone treatments have shown only limited efficacy. In contrast, tyrosine kinase inhibitors and monoclonal antibodies, especially those targeting angiogenic signaling such as sunitinib and bevacizumab, have shown promising antitumoral activity in small phase 2 trials. Moreover, results of recent landmark studies on (epi-)genetic alterations in meningioma revealed potential therapeutic targets which are currently under investigation. These include inhibitors of mammalian target of rapamycin (mTOR), focal adhesion kinase (FAK), cyclin-dependent kinases (CDK), phosphoinositide-3-kinase (PI3K), sonic hedgehog signaling, and histone deacetylases. In addition, clinical trials evaluating immune checkpoint inhibitors such as ipilimumab, nivolumab, pembrolizumab and avelumab are currently being conducted and early results suggest clinically meaningful responses in a subset of patients.

**Conclusions:**

There is a paucity of high-level evidence on systemic treatment options in meningioma. However, interesting novel treatment targets have been identified in the last decade. Positive signals of anti-angiogenic agents, genomically targeted agents and immunotherapy in early phase trials should be confirmed in large prospective controlled trials.

## Introduction

Meningiomas are the most common primary central nervous system (CNS) neoplasms in adults. Accounting for 39% of tumors in the CNS, their incidence reaches about 9.1/100.000 person-years in the United States, with a predominance in female individuals of higher age [1]. In the recent update of the WHO Classification of Central Nervous Tumours in 2021, meningiomas are classified into CNS WHO grades 1–3 according to histopathological features such as the number of mitotic figures, invasive growth pattern, specific morphological subtypes and anaplastic features but also genetic characteristics such as telomerase reverse transcriptase (*TERT*) promoter mutations or homozygous deletions of *CDKN2A/2B* [2]. Whereas CNS WHO grade 1 meningiomas grow slowly and in a well-demarcated pattern, atypical (CNS WHO grade 2) or anaplastic (CNS WHO grade 3) meningiomas may show malignant characteristics such as rapid growth or brain invasion. Although CNS WHO grade 2 and 3 meningiomas occur in only 4–28% and 1–3% of cases [3, 4], they represent a high clinical need as they show higher recurrence rates after resection [5] and may metastasize extracranially to the lungs, liver, or bones [6].

According to current guidelines [7, 8], asymptomatic meningiomas with no mass effect can be followed by a watch-and-wait approach with annual magnetic resonance imaging (MRI). However, growing and/or symptomatic meningiomas with mass effect should be treated by maximal safe resection with curative intent. Indeed, extent of resection has been repeatedly shown as a prognostic factor, with higher recurrence rates and worse survival in higher-grade meningioma [9, 10]. Therefore, radiotherapy or stereotactic radiosurgery should be considered in meningiomas that were not gross totally resected (GTR) as well as higher-grade tumors. The role of systemic therapy remains unclear due to a lack of evidence, and pharmacological treatment of meningiomas is generally regarded as experimental. However, systemic treatment options are frequently used as salvage treatment in situations where no further local therapeutic options are available. Overall, cytotoxic agents have shown limited activity, whereas targeted treatment approaches, especially anti-angiogenic agents, have shown some efficacy in the salvage treatment of meningioma [7]. Here, we aim to summarize the available evidence on systemic treatment options in meningioma and provide an overview of currently studied agents and future prospects.

## Clinical trial endpoints and assessment of therapy response in meningioma

Meningiomas are heterogeneous tumors in terms of growth rate, clinical course and therefore prognosis. Consequently, the definition of appropriate clinical trial endpoints and response criteria remains challenging, and recommendations for response criteria and clinical trial endpoints were issued only recently [11]. While overall survival (OS) is generally regarded as the primary benchmark to evaluate the efficacy of anticancer treatments, use of this parameter is complicated by the long follow-up times, especially in relatively benign tumors such as CNS WHO grade 1 meningiomas. As valid historical data are missing, use of OS as a clinical trial endpoint is only reasonable in randomized trials with a respective control arm. Radiological response parameters such as objective response rate (ORR) are also used; however, clearly defined radiological assessment criteria were lacking in meningioma. This also complicates the use of progression-free survival (PFS) and PFS rates, as progression may be defined differently between trials and historical controls. Moreover, progression may be easily overlooked due to the slow growth rate of most meningiomas. Still, PFS and PFS rates are a frequently used surrogate parameter for assessing the activity of a treatment without considering the potential impact of post-progression treatments. In addition, the absence of meningioma progression may also best reflect clinical stability in terms of neurological symptom burden. Indeed, most clinical trials reported PFS at 6 months (PFS-6), providing a large number of historical controls of meningiomas of all grades.

## Systemic treatment in meningioma – the status quo

The efficacy of cytotoxic agents such as hydroxyurea [12–15], irinotecan [16], temozolomide [17, 18], or combination regimens such as vincristine, adriamycin and cyclophosphamide (VAC) [19] has been evaluated, with overall limited efficacy (Table 1). Whereas the DNA-intercalating agent trabectedin has shown promising activity in vitro and in one heavily pretreated patient [20], a prospective randomized phase 2 trial (EORTC 1320) failed to meet its primary endpoint, with no difference to physician’s choice in terms of antitumoral activity but significantly higher toxicity [21].

**Table 1:** Selected evidence for systemic treatment options in meningioma

Drug	Drug class	Main inclusion criterion	Number of patients	Clinical phase	Outcome data	Ref
Hydroxyurea	Cytotoxic	Recurrent WHO grade 2–3 meningioma	n = 35	Retrospective case series	PFS-6: 3% Median PFS 2 months (95%CI 1.6–2.4)	[12]
		Recurrent WHO grade 1–2 meningioma	n = 12	Prospective, phase not stated	Median time to progression (TTP): 13 months (range: 2–24)	[13]
		Recurrent or unresectable WHO grade 1–3 meningioma	n = 20	Prospective, phase not stated	PFS-12: 93% PFS-24: 77%	[14]
		Recurrent WHO grade 1–3 meningioma	n = 4	Retrospective case series	No aggregated data given	[15]
		Recurrent WHO grade 2–3 meningioma	n = 13	Phase 2 (unplanned post-hoc analysis)	PFS-6: 8.8% Median PFS: 2.4 months (95%CI: 1.4–4.2) OS-6: 55.9% Median OS: 7.4 months (95%CI: 3.1–19.9)	[21]
Irinotecan	Cytotoxic	Recurrent WHO grade 1 meningioma	n = 16	Phase 2	PFS-6: 6% Median OS: 7 months	[16]
Temozolomide	Cytotoxic	WHO grade 1–3 meningioma receiving radiotherapy	n = 11	Retrospective case series	PFS-6: 91.7%	[17]
		Recurrent WHO grade 1 meningioma	n = 16	Phase 2	Median TTP: 5 months (range: 2.5–5)	[18]
Vincristine, adriamycin, cyclophosphamide (VAC)	Cytotoxic	Treatment-naïve WHO grade 3 meningioma	n = 14	Phase 2	Median TTP: 4.6 years (range: 2.2–7.1) Median OS: 5.3 years (range: 2.6–7.6)	[19]
Trabectedin	Cytotoxic	Recurrent WHO grade 2–3 meningioma	n = 90 (trabectedin: n = 61; local standard of care: n = 29)	Phase 2 (local standard of care as control arm)	Median PFS: 2.43 (trabectedin) vs. 4.17 months (local standard of care) PFS-6: 21.1% (trabectedin) vs. 29.1% (local standard of care) Median OS: 11.73 (trabectedin) vs. 10.61 months (local standard of care)	[21]
Octreotide	Somatostatin analog	Recurrent WHO grade 1–3 meningioma or meningeal hemangiopericytoma	n = 12	Phase 2	Median TTP: 17 weeks Median OS: 2.7 years (range: 22 days to 9.4 years)	[23]
		Recurrent WHO grade 2–3 meningioma	n = 9	Phase 2	Median TTP: 4.23 months PFS-6: 44.4%	[25]
Pasireotide	Somatostatin analog	Recurrent WHO grade 1–3 meningioma	n = 34	Phase 2	WHO grade 1: - PFS-6: 50%, median PFS: 26 weeks (95%CI 12–43) WHO grade 2–3: - PFS-6: 17%, median PFS: 15 weeks (95%CI 8–20)	[24]
Octreotide + everolimus	Somatostatin analog + mTOR inhibitor	Recurrent WHO grade 1–3 meningioma	n = 20	Phase 2	PFS-6: 55% (95%CI: 31.1%-73.5%) OS-6: 90% (95%CI: 65.6%-97.4%) Decrease > 50% in tumor size in 78% of tumors	[27]
^90^Y-DOTATOC, ^177^Lu-DOTATOC	Radionucleid-somatostatin analog conjugate	Recurrent and unresectable WHO grade 1–3 meningioma	n = 34	Phase 2	Mean OS: 8.6 years	[26]
Sunitinib	Multi-tyrosine kinase inhibitor (VEGFR, PDGFR)	Recurrent WHO grade 2–3 meningioma	n = 36	Phase 2	PFS-6: 42% Median PFS: 5.2 months (95%CI: 2.8–8.3) Median OS: 24.6 months (95%CI: 16.5–38.4)	[31]
Vatalanib	Multi-tyrosine kinase inhibitor (VEGFR, PDGFR, c-kit)	Recurrent radiation- and surgery-refractory WHO grade 1–3 meningioma	n = 25	Phase 2	WHO Grade 2: - PFS-6: 64.3% - Median PFS: 6.5 months - Median OS: 26.0 months WHO Grade 3: - PFS-6: 37.5%% - Median PFS: 3.6 months - Median OS: 23 months	[33]
Bevacizumab	Monoclonal anti-VEGF antibody	WHO grade 2–3 meningioma	n = 15	Retrospective case series	PFS-6: 43.8% Median PFS: 26 weeks (95%CI: 10–29 weeks)	[34]
		Recurrent WHO grade 1–3 meningioma	n = 14	Retrospective case series	PFS-6: 86% Median PFS: 17.9 months (95%CI: 8.5 – not reached) Median OS: not reached	[35]
		Recurrent WHO grade 2–3 meningioma	n = 9	Phase 2 (unplanned post-hoc analysis)	PFS-6: 44.4% Median PFS: 6 months (95%CI: 2.1–18.6) OS-6: 88.9% Median OS: 13.5 months (95%CI: 5.4-not reached)	[21]
Bevacizumab + everolimus	Monoclonal anti-VEGF antibody + mTOR inhibitor	Recurrent WHO grade 1–3 meningioma	n = 17	Phase 2	PFS-6: 69% Median PFS: 22 months (95%CI: 4.5–26.8) Median OS: 23.8 months (95%CI: 9.0–33.1)	[36]
Imatinib	Multi-tyrosine kinase inhibitor (PDGFR, c-kit, Bcr-abl)	Recurrent WHO grade 1–3 meningioma	n = 23	Phase 2	PFS-6: 29.4% Median PFS: 2 months (range: 0.7–34)	[39]
Imatinib + hydroxyurea	Multi-tyrosine kinase inhibitor (PDGFR, c-kit, Bcr-abl) + cytotoxic agent	Recurrent WHO grade 1–3 meningioma	n = 21	Phase 2	PFS-6: 61.9% Median PFS: 7.0 months (95%CI: 2.8–9.2) Median OS: 66.0 months (95%CI: 20.7–66.0)	[38]
		Recurrent WHO grade 1–3 meningioma	n = 15 (imatinib + hydroxyurea: 7 patients; hydroxyurea alone: 8 pts)	Phase 2	Imatinib + hydroxyurea: - PFS-9: 0% - Median PFS: 4 months Hydroxyurea: - PFS-9: 75% - Median PFS: 19 months	[40]
Erlotinib or gefitinib	Tyrosine kinase inhibitor (EGFR)	Recurrent WHO grade 1–3 meningioma	n = 25 (erlotinib: n = 9; gefitinib: n = 16)	Phase 2 (post-hoc analysis in pilot component of glioma trial)	Whole cohort: - PFS-6: 28% - Median PFS: 10 weeks (95%CI: 8–20) - OS-6: 76% - Median S: 23 months (95%CI: 11-not reached)	[41]
Vistusertib	mTOR inhibitor	Progressive or symptomatic meningiomas in patients with neurofibromatosis 2	n = 18	Phase 2	PFS-6: 88.9% Median PFS: not reached (95%CI: 24–not reached)	[45]
AR-42	Histone deacetylase inhibitor	NF2-associated vestibular schwannoma and meningioma and sporadic meningioma	n = 7	Phase 1 (post-hoc analysis of phase 1 trial in advanced solid tumors)	No aggregated data, slowed tumor growth	[66]
IFN-α	Cytokine	Recurrent WHO grade 1 meningioma	n = 35	Phase 2	PFS-6: 54% PFS-12: 31% Median TTP: 7 months (range: 2–24) Median OS: 8 months (range: 3–28)	[67]
		Recurrent WHO grade 2–3 meningioma	n = 35	Retrospective case series	PFS-6: 17% (95%CI: 7–31%) Median PFS: 12 weeks (95%CI: 8–20 weeks)	[70]
Pembrolizumab	Monoclonal anti-PD-1 antibody	Recurrent and progressive WHO grade 2–3 meningioma	n = 25	Phase 2	PFS-6: 48% (90%CI: 31–66%) Median PFS: 7.6 months (90%CI: 3.4–12.9)	[84]

As many meningiomas show overexpression of the somatostatin receptor 2A [22], somatostatin analogs such as octreotide or pasireotide [23–25] as well as targeted radionucleotide therapy have also been studied [26], with varying degrees of efficacy. Moreover, a phase 2 trial evaluating the combination of octreotide with the mammalian target of rapamycin (mTOR) everolimus has shown clinical activity and a decreased growth rate in WHO grade 1–3 meningioma [27, 28]. Similarly, due to the high expression of progesterone receptor on meningioma cells, the progesterone antagonist mifepristone has been considered among other hormonal agents, although no clinically meaningful activity was demonstrated [29].

More promising results have been observed with tyrosine kinase inhibitors, especially those targeting angiogenic pathways such as vascular endothelial growth factor (VEGF) signaling. Indeed, soluble isoforms of VEGF have been detected in WHO grade 2 and 3 meningiomas which also showed higher microvascular density as compared to WHO grade 1 tumors [30]. These results suggest that VEGF-directed agents could be reasonable agents for the management of higher-grade meningiomas. Consistent with this hypothesis, a phase 2 trial of sunitinib in 36 patients with atypical and anaplastic meningioma showed a progression-free survival (PFS) rate of 42% at 6 months (PFS-6), comparing well with historical controls [31, 32]. Similar results have been observed with the tyrosine kinase inhibitor vatalanib (PTK787) which targets VEGF signaling, platelet-derived growth factor receptor (PDGFR), and c-kit [33]. A small retrospective series of 15 patients with atypical or anaplastic meningioma treated with bevacizumab found a median PFS of 26 weeks and a PFS-6 of 43.8% [34]. Another retrospective study showed a PFS-6 of even 86%, with no significant improvement if cytotoxic chemotherapy was added [35]. Similar results were seen in a small phase 2 trial of a combination treatment consisting of bevacizumab and the mTOR inhibitor everolimus [36]. Bevacizumab was also associated with growth-inhibitory and anti-edematous activity in longitudinal imaging analyses [37]. Other previously studied drugs include imatinib, erlotinib and gefitinib, with no relevant clinical activity [38–41].

These results which mainly stem from retrospective or small prospective studies could be substantiated in exploratory analyses of the EORTC 1320 study, where physician’s choice was included as a control arm [21]. Control treatments included the cytotoxic compounds hydroxyurea, vincristine, cyclophosphamide, doxorubicin as well as bevacizumab and somatostatin analogs. An unplanned post-hoc analysis corroborated the relative superiority of bevacizumab (median PFS: 6 months, PFS-6: 44.4%) over hydroxyurea (median PFS: 2.4 months, PFS-6: 8.8%) and over the experimental drug trabectedin (median PFS: 2.4 months, PFS-6: 24.4%). However, these were unpowered analyses, and further prospective trials are needed to clarify the efficacy of bevacizumab and other anti-angiogenic agents in meningioma.

## Frequent genetic alterations and potential therapeutic implications

**Fig. 1:**
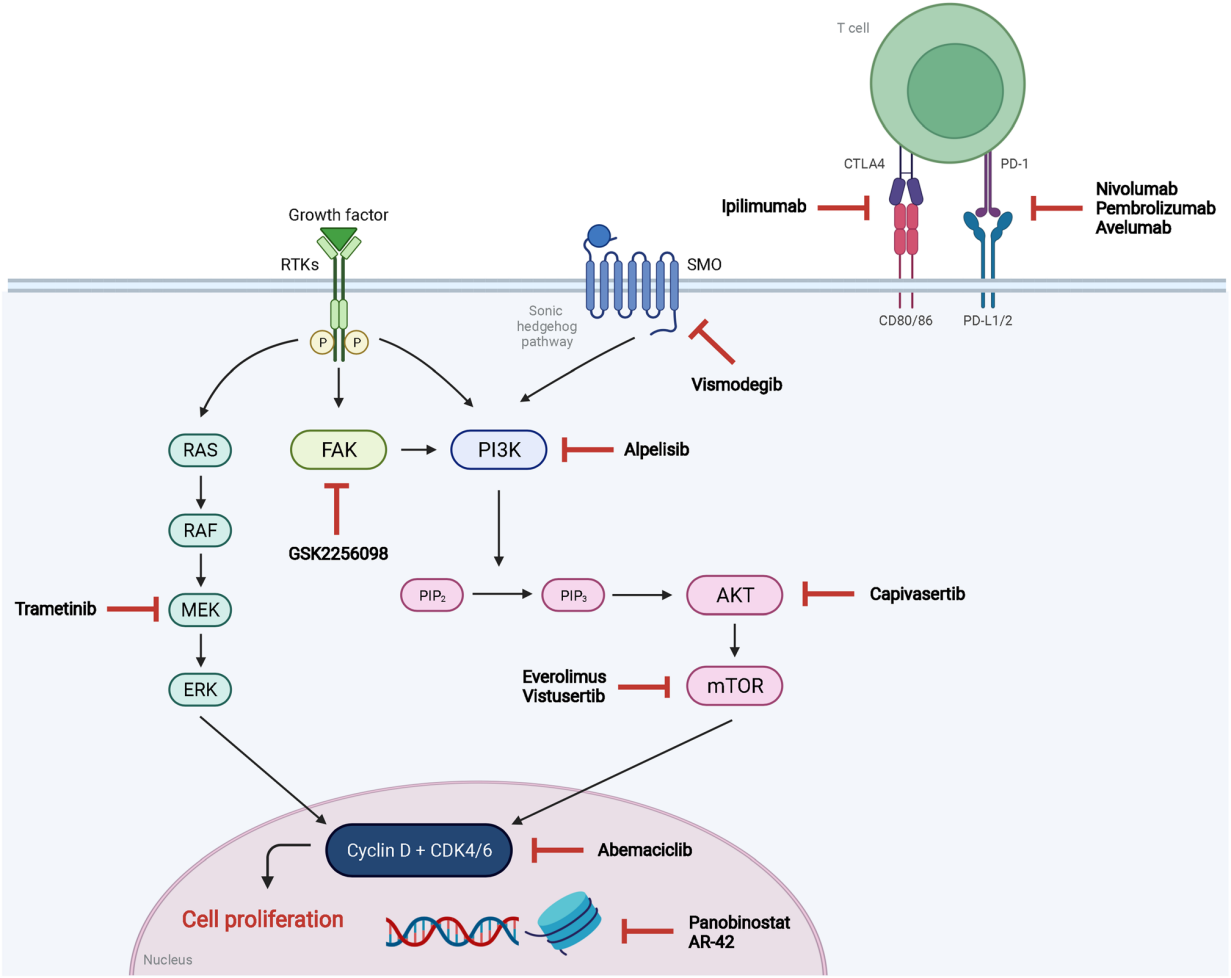
Emerging targets and candidate drugs of systemic treatment in meningioma. Abbreviations are given in text

Based on data of high-throughput landmark studies, significant advances have been made concerning the genetic signature and molecular pathogenesis of meningiomas of different grades and tumor locations [42, 43]. For an in-depth review on this topic, we refer to the review of Preusser et al. [44]. Here, we summarize the available knowledge on frequent alterations and discuss their potential as targets for novel systemic treatment options based on results of preclinical and planned early phase clinical studies (Fig. 1, Table 2).

**Table 2:** Selection of ongoing clinical trials of systemic therapy in meningioma

Clinical trial identification	Drug	Drug class	Main inclusion criterion	Status (effective 12/07/2022)
NCT03071874 (phase 2)	Vistusertib	mTOR inhibitor	Recurrent or progressing WHO grade 2–3 meningioma	Active, not recruiting
NCT02523014 (phase 2)	- GSK2256098 - Abemaciclib - Capivasertib - Vismodegib	- FAK inhibitor - CDK4/6 inhibitor - AKT inhibitor - SHH inhibitor	Progressing WHO grade 1–3 meningioma	Recruiting
NCT03631953 (phase 1)	Alpelisib + trametinib	PI3K inhibitor + MEK inhbitor	Progressing WHO grade 1–3 meningioma	Recruiting
NCT03220646 (phase 2)	Abemaciclib	CDK4/6 inhibitor	Recurrent primary brain tumors of all grades (including glioma, meningioma, ependymoma, primary central nervous system lymphoma)	Active, not recruiting
NCT01324635 (phase 1)	Panobinostat (+ stereotactic radiation)	Histone deacetylase inhibitor	Recurrent gliomas, high-grade meningiomas, and brain metastases	Terminated, no results published
NCT03604978 (phase 1/2)	Nivolumab ± ipilimumab (+ stereotactic radiosurgery)	monoclonal anti-PD-1 and anti-CTLA-4 antibodies	Recurrent WHO grade 2–3 meningioma	Recruiting
NCT02648997 (phase 2)	Nivolumab monotherapy (cohort 1) Nivolumab + ipilimumab after radiation (cohort 2)	monoclonal anti-PD-1 and anti-CTLA-4 antibodies	Recurrent/progressive WHO grade 2–3 meningioma	Recruiting
NCT03173950 (phase 2)	Nivolumab	monoclonal anti-PD-1 antibody	Recurrent rare primary brain tumors (including WHO grade 2–3 meningioma, medulloblastoma, ependymoma, pineal region and choroid plexus tumors)	Recruiting
NCT04659811 (phase 2)	Pembrolizumab (+ stereotactic radiosurgery)	monoclonal anti-PD-1 antibody	Recurrent WHO grade 1–3 meningioma	Recruiting
NCT03279692 (phase 2)	Pembrolizumab	monoclonal anti-PD-1 antibody	Recurrent WHO grade 2–3 meningioma	Active, not recruiting
NCT03016091 (phase 2)	Pembrolizumab	monoclonal anti-PD-1 antibody	Recurrent WHO grade 2–3 meningioma or hemangiopericytoma	Unknown
NCT03267836 (phase 1)	Avelumab (neoadjuvant in combination with proton radiation therapy followed by surgery)	monoclonal anti-PD-L1 antibody	Recurrent or progressive WHO grade 1–3 meningioma	Active, not recruiting

### Neurofibromin 2/Merlin

Nearly half of sporadic meningiomas carry loss-of-function mutations in the tumor suppressor gene *NF2* encoding for the protein Merlin [43]. Conversely, patients with neurofibromatosis type 2 carrying germline mutations in *NF2* have a significantly higher risk for meningioma in their lifetime and even during childhood [44]. From a pathogenetic point of view, the encoded protein Merlin has an inhibitory role on the growth-promoting phosphoinositide 3-kinase (PI3K)/AKT/mTOR pathway, providing a potential treatment target as this pathway might be constitutively activated in the presence of *NF2* mutations. Whereas data on the efficacy of everolimus had been published previously [36], the mTOR inhibitor vistusertib (AZD2014) has also been investigated. A phase 2 trial assessed vistusertib in 18 patients with WHO grade 2–3 meningioma (NCT02831257), with a promising PFS-6 of 88.9% according to early data [45]. Another phase 2 trial of vistusertib in WHO grade 2–3 meningioma is ongoing (NCT03071874). As another potential target, focal adhesion kinase (FAK) inhibition has been shown to exert antitumoral activity in in vitro meningioma models with *NF2* loss [46]. In line, the FAK inhibitor GSK2256098 is being evaluated in patients with progressive meningioma in a still recruiting multi-arm phase 2 trial (Alliance A071401, NCT02523014) along with the cyclin-dependent kinase 4/6 inhibitor abemaciclib, the AKT inhibitor capivasertib, and the sonic hedgehog (SHH) inhibitor vismodegib. Early results of FAK inhibition in recurrent or progressive meningioma have shown a PFS-6 of 83.3% and a median PFS of 12.8 months in WHO grade 1 meningioma, whereas PFS-6 was 33.3% and median PFS 3.7 months in WHO grade 2–3 meningioma. GSK2256098 was generally well tolerated [47].

### Tumor necrosis factor receptor-associated factor 7 (TRAF7) and Krupple-like factor 4 (KLF4)

*TRAF7* mutations occur in ~ 25% of meningiomas and seem to be mutually exclusive with *NF2* mutations according to a genomic landmark study of 300 meningiomas [42]. Functionally, TRAF7 is a ubiquitin ligase impacting a variety of signaling pathways including NF-κB, the MAP kinase pathway, among others, and has physiologically a pro-apoptotic function [44]. Likewise, *KLF4* mutations seem to occur only in *NF2*-intact meningiomas and frequently co-exist with alterations of *TRAF7* [42]. Physiologically, the encoded protein KLF4 is involved in stem cell renewal and differentiation. Both *TRAF7* and *KLF4* alterations in meningioma are loss-of-function mutations and therefore not directly targetable. Thus, further research is needed to elucidate the pathogenetic implications of these mutations and identify potentially druggable downstream targets. Of note, KLF4-mutated meningiomas exhibit higher sensitivity to mTOR inhibitors such as temsirolimus [48], underlining the potential role of the PI3K/AKT/mTOR pathway as potential treatment target in meningioma.

### AKT serine/threonine kinase 1 (AKT1) and phosphoinositide-3-kinase (PI3K)

The *AKT1* E17K mutation is a known oncogenic alteration which was detected in about 8–13% of meningiomas [42, 43], especially those located in the skull base where 31% of tumors were found to display this alteration [49, 50]. Indeed, this specific mutation occurs in a small subset of breast, uterine, ovarian, cervical, lung, prostate, as well as colorectal cancers, and specific inhibitors such as capivasertib are under investigation [51]. In the above-mentioned multi-arm phase 2 trial (NCT02523014) of patients with progressive *NF2-*altered meningioma, capivasertib has been included as one of four experimental treatments for patients with AKT mutant meningiomas. Similar to *NF2* and *KLF4*, *AKT* mutations lead to a functional upregulation of the PI3K/AKT/mTOR pathway. In addition, also mutations of the gene encoding for the PI3K catalytic subunit alpha (*PIK3CA)* have been found in about 7% of non-*NF2*-altered meningiomas [52]. *PIK3CA* mutations are also known in other solid tumors such as breast cancer where the PI3K inhibitor alpelisib is currently approved for treatment hormone receptor-positive, HER2-negative disease with progression after first-line therapy [53]. The combination of alpelisib and the MEK inhibitor trametinib is currently studied in a phase 1 trial in progressive refractory meningioma (NCT03631953) based on unpublished preclinical results that trametinib may induce apoptosis in meningioma cell lines. Like *TRAF7/KLF4*-mutated meningiomas, also *AKT1*-mutated tumors are frequently found in the skull base. As these lesions are characterized by a comparably favorable prognosis, the feasibility of clinical trials in these meningiomas is limited by the relatively low occurrence of clinically relevant tumor progression.

### Smoothened, frizzle class receptor (SMO)

*SMO* mutations occur in about 5% of meningiomas which do not show alterations in *NF2, AKT1 and KLF1* [42, 43]. The encoded protein is a receptor activating the sonic hedgehog signaling (SHH) pathway which is involved in multiple cellular processes such as differentiation and proliferation. Alterations have been described in a wide array of solid tumors including breast cancer, pancreatic cancer, colorectal cancer, gastric cancer, hepatocellular cancer, cholangiocarcinoma, lung cancer, and medulloblastoma [54]. Moreover, the SHH pathway is involved in the pathogenesis of basal cell carcinoma, where the specific inhibitor vismodegib is approved in Europe and the US. Vismodegib is being evaluated in the multi-arm trial described above (NCT02523014) in progressive meningioma. However, a recent publication suggests that *SMO* mutations may not be associated with an activation of the SHH pathway in preclinical models of meningioma, potentially challenging the efficacy of vismodegib in these tumors [55].

### Cyclin-dependent kinase inhibitor 2A/B (CDKN2A/B)

With the recent 2021 update of the WHO Classification of Central Nervous System Tumours, homozygous deletions of *CDKN2A/B* are sufficient to designate meningiomas as CNS WHO grade 3 tumors regardless of histological grading [2]. Previously, *CDKN2A/B* alterations had been mainly described in anaplastic meningiomas [56]. Meningiomas harboring homozygous deletions of *CDKN2A/B* are characterized by high recurrence rates independently from WHO grade, DNA methylation class, sex, age and tumor location [57]. In addition, also heterozygous loss, mutations, and promoter methylation of *CDKN2A* was found to be strongly related to recurrent meningiomas and a high Ki-67 index [56]. Physiologically, the proteins encoded by *CDKN2A/B* halt the cell cycle; consequently, homozygous loss leads to dysregulated cell cycle progression and uncontrolled proliferation.

Pharmacological inhibition of cyclin-dependent kinases CDK4/6 could represent a particularly promising strategy in higher-grade meningiomas with high mitotic activity independently from *CDKN2A/B* status. The CDK4/6 inhibitors palbociclib, ribociclib and abemaciclib are approved for use in hormone receptor-positive breast cancer in combination with endocrine therapy. In preclinical models of meningioma, palbociclib with radiation has shown decreased proliferation and in vivo tumor size [58]. However, data from clinical trials are to be awaited. Currently, the multi-arm trial NCT02523014 is evaluating abemaciclib in recurrent meningioma harboring CDK pathway or *NF2* alterations. Moreover, abemaciclib is being assessed in a tissue-agnostic phase 2 trial in patients with recurrent brain tumors (NCT03220646).

## The epigenetic landscape of meningioma as potential treatment target?

Analysis of the DNA methylome is increasingly being used as an additional tool in the diagnosis of CNS malignancies as it defines biologically homogenous subgroups [59]. In meningioma, a large study based on 497 samples has revealed six distinct methylation clusters (benign 1–3, intermediate A/B and malignant) which also correlated with clinical factors such as sex, tumor location and prognosis [60, 61]. Another publication defined a prognostically relevant methylation signature, where certain CpG sites displayed a higher degree of methylation in tumors of patients with worse survival [62]. In addition, some meningiomas show mutations in KDM5C, KDM6A, SMARCB1, and SMARCE1 which encode for histone demethylases (KDM5C, KDM6A) or proteins involved in transcription-related chromatin remodeling (SMARCB1, SMARCE1) [21, 42]. Based on these results, epigenetic modification could represent a novel therapeutic approach. Indeed, the histone deacetylase (HDAC) inhibitor vorinostat showed activity in ex vivo models of tumors with a specific molecular pattern based on DNA methylation analysis, RNA sequencing, whole-exome sequencing and copy number alterations [63]. Moreover, in *NF2*-altered preclinical meningioma models, the HDAC inhibitor AR-42 showed some antitumoral activity [64, 65]. These results were evaluated in a phase 1 pilot trial of AR-42 in *NF2*-associated vestibular schwannomas and meningiomas [66] with mixed results, but further data are needed. In this regard, a phase 1 trial is currently evaluating the histone deacetylase inhibitor panobinostat with stereotactic radiation in patients with high-grade meningioma, recurrent glioma and brain metastases (NCT01324635).

## Immune-modulating approaches

In the last decades, the cytokine interferon alpha (IFN-α) has been evaluated as potential treatment option in meningioma. Indeed, case reports and small clinical trials have suggested antitumoral activity of IFN-α [67, 68]. IFN-α likely exerts a antiproliferative activity, but antiangiogenic and immune-modulatory properties have also been postulated [69]. However, another retrospective case series failed to show clinically meaningful efficacy in higher-grade meningioma [70].

Immune checkpoint inhibitors (ICI) have revolutionized the treatment of solid tumors, as durable responses can be observed in metastatic disease across various histologies with previously dismal prognosis. ICI targeting the programmed death receptor (ligand) 1 (PD-1/PD-L1) and cytotoxic T-lymphocyte-associated protein 4 (CTLA-4) axis are widely applied in solid malignancies such as melanoma, lung cancer, renal cell carcinoma, head and neck squamous cell carcinoma, among others. Whereas ICI have shown activity in asymptomatic patients with brain metastases [71–74], clinical trials have failed to show an overall benefit in primary CNS malignancies such as glioblastoma both in newly diagnosed disease as well as in the recurrent setting [75–77].

PD-L1 expression is frequently used as a biomarker predicting the response towards ICI. However, previous studies on PD-L1 expression in meningioma are conflicting. Membranous PD-L1 expression was found in ~ 5 to > 80% of meningeal neoplasms, with higher expression in higher-grade tumors and mainly on myeloid cells within the tumor microenvironment [78–81]. In anaplastic meningioma, an elevated density of FOXP3 + infiltrating lymphocytes was seen, suggesting a prime role of regulatory T cells in the particularly immunosuppressive microenvironment in higher-grade meningioma [81–83]. With regard to clinical trials, the results of a phase 2 trial evaluating pembrolizumab in WHO grade 2 and 3 meningiomas at recurrence or progression have been published recently, demonstrating that the trial met its primary endpoint [84]. PFS-6 reached 48%, while median PFS was 7.6 months, with 10 out of 25 patients still being alive at database lock. Moreover, clinical responses were also observed in metastatic or extracranial disease. Biomarker studies have also been included, with an observed trend for a correlation of clinical benefit with PD-L1 expression and apparent diffusion coefficients (ADC) as evaluated in magnetic resonance imaging. Further prospective studies will be needed to validate these results and define predictive biomarkers allowing for a rational selection of patients with meningioma who might benefit from ICI.

Other trials of ICI in meningeal neoplasms are ongoing. Two NCI-sponsored phase 2 trials aim to evaluate nivolumab ± ipilimumab with stereotactic radiosurgery or external beam radiotherapy in recurrent WHO grade 2–3 meningiomas (NCT03604978, NCT02648997). Another study is assessing nivolumab alone in recurrent rare CNS malignancies including WHO grade 2–3 meningioma, ependymoma, pineal region tumors, medulloblastoma, and choroid plexus tumors (NCT03173950). Similar trials are evaluating the ICIs pembrolizumab (NCT04659811, NCT03279692, NCT03016091) and avelumab (NCT03267836).

Moreover, the myeloid cell compartment is increasingly considered as an emerging treatment target, as tumor-associated myeloid cells stimulate tumor growth by secreting growth-promoting factors. Inhibiting chemotactic signals which are responsible for the recruitment of myeloid cells to the tumor microenvironment could therefore represent an interesting therapeutic strategy, especially in tumors such as meningiomas which are abundantly infiltrated by myeloid cells hampering antitumoral immune responses. One of these signals is the colony-stimulating factor 1 (CSF-1) axis. Indeed, a recent study by Yeung et al. showed a high expression of CSF-1 receptor on macrophages within the meningioma microenvironment, and treatment with monoclonal antibodies targeting this signaling pathway was associated with decreased meningioma growth in murine models [85].

## Conclusion and future prospects

Recurrent meningiomas which are not amenable for local treatment options such as surgery or radiotherapy remain a therapeutic challenge. Whereas systemic treatments are frequently considered in these situations, the evidence for their use is overall scarce as controlled trials are rare and historical benchmark data on the outcome of higher-grade meningiomas are limited. Traditional cytotoxic agents are generally ineffective. However, preclinical data suggest antitumoral activity of the antimetabolite gemcitabine, but clinical trials are pending [86]. Antiangiogenic therapies such as multi-tyrosine kinase inhibitors or antibodies targeting the VEGF axis showed promising results in small phase 2 trials and retrospective case series. However, prospective controlled trials are urgently needed to validate these positive findings. In addition, the elucidation of the (epi-)genetic landscape of glioma by high-throughput landmark studies has revealed further potential therapeutic targets which are currently under investigation. Recent genomic studies have identified novel potential targets, which are being evaluated in ongoing national studies. Immunotherapeutic approaches including ICI are also being evaluated, and early results suggest a promising activity in a subset of patients.

## Data Availability

Not applicable.
